# A microbial platform for renewable propane synthesis based on a fermentative butanol pathway

**DOI:** 10.1186/s13068-015-0231-1

**Published:** 2015-04-10

**Authors:** Navya Menon, András Pásztor, Binuraj RK Menon, Pauli Kallio, Karl Fisher, M Kalim Akhtar, David Leys, Patrik R Jones, Nigel S Scrutton

**Affiliations:** BBSRC/EPSRC Centre for Synthetic Biology of Fine and Speciality Chemicals, Manchester Institute of Biotechnology, Faculty of Life Sciences, 131 Princess Street, The University of Manchester, Manchester, M1 7DN UK; Molecular Plant Biology, Department of Biochemistry, Tykistökatu 6A 6krs, University of Turku, FI 20014 TURUN YLIOPISTO, Turku, Finland; Department of Life Sciences, Imperial College London, Sir Alexander Fleming Building, South Kensington Campus, London, SW7 2AZ UK

**Keywords:** Propane, Butanol, Microbial pathway engineering, Cyanobacteria, Aldehyde deformylating oxygenase, *Escherchia coli*

## Abstract

**Background:**

Propane (C_3_H_8_) is a volatile hydrocarbon with highly favourable physicochemical properties as a fuel, in addition to existing global markets and infrastructure for storage, distribution and utilization in a wide range of applications. Consequently, propane is an attractive target product in research aimed at developing new renewable alternatives to complement currently used petroleum-derived fuels. This study focuses on the construction and evaluation of alternative microbial biosynthetic pathways for the production of renewable propane. The new pathways utilize CoA intermediates that are derived from clostridial-like fermentative butanol pathways and are therefore distinct from the first microbial propane pathways recently engineered in *Escherichia coli*.

**Results:**

We report the assembly and evaluation of four different synthetic pathways for the production of propane and butanol, designated a) *atoB*-*adhE2* route, b) *atoB*-*TPC7* route, c) *nphT7*-*adhE2* route and d) *nphT7*-*TPC7* route. The highest butanol titres were achieved with the *atoB-adhE2* (473 ± 3 mg/L) and *atoB-TPC7* (163 ± 2 mg/L) routes. When aldehyde deformylating oxygenase (ADO) was co-expressed with these pathways, the engineered hosts also produced propane. The *atoB-TPC7-ADO* pathway was the most effective in producing propane (220 ± 3 μg/L). By (i) deleting competing pathways, (ii) including a previously designed ADO_A134F_ variant with an enhanced specificity towards short-chain substrates and (iii) including a ferredoxin-based electron supply system, the propane titre was increased (3.40 ± 0.19 mg/L).

**Conclusions:**

This study expands the metabolic toolbox for renewable propane production and provides new insight and understanding for the development of next-generation biofuel platforms. In developing an alternative CoA-dependent fermentative butanol pathway, which includes an engineered ADO variant (ADO_A134F_), the study addresses known limitations, including the low bio-availability of butyraldehyde precursors and poor activity of ADO with butyraldehyde.

**Graphical abstract:**
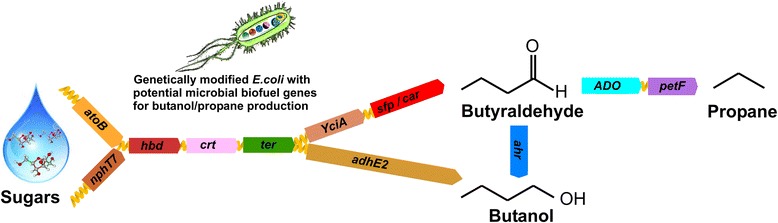
Propane synthesis derived from a fermentative butanol pathway is enabled by metabolic engineering.

**Electronic supplementary material:**

The online version of this article (doi:10.1186/s13068-015-0231-1) contains supplementary material, which is available to authorized users.

## Background

Propane, a major component of autogas or liquefied petroleum gas (LPG), is an emerging fuel for future energy supply and transportation [1]. Propane is the third most widely used motor fuel and about 20 million tonnes of propane gas are used per year to fuel motor vehicles [2]. It is estimated that propane provides heat and energy for more than 14 million homes worldwide annually [3,4]. Propane also has an existing global market for a wide number of other stationary and mobile applications, such as low emission vehicles, gas burners and refrigeration systems [5]. Easy separation from liquid biotechnological processes as a gas and less energy requirements for liquefaction and storage offers potential advantages to propane over other gaseous fuels [6].

**Figure 1:**
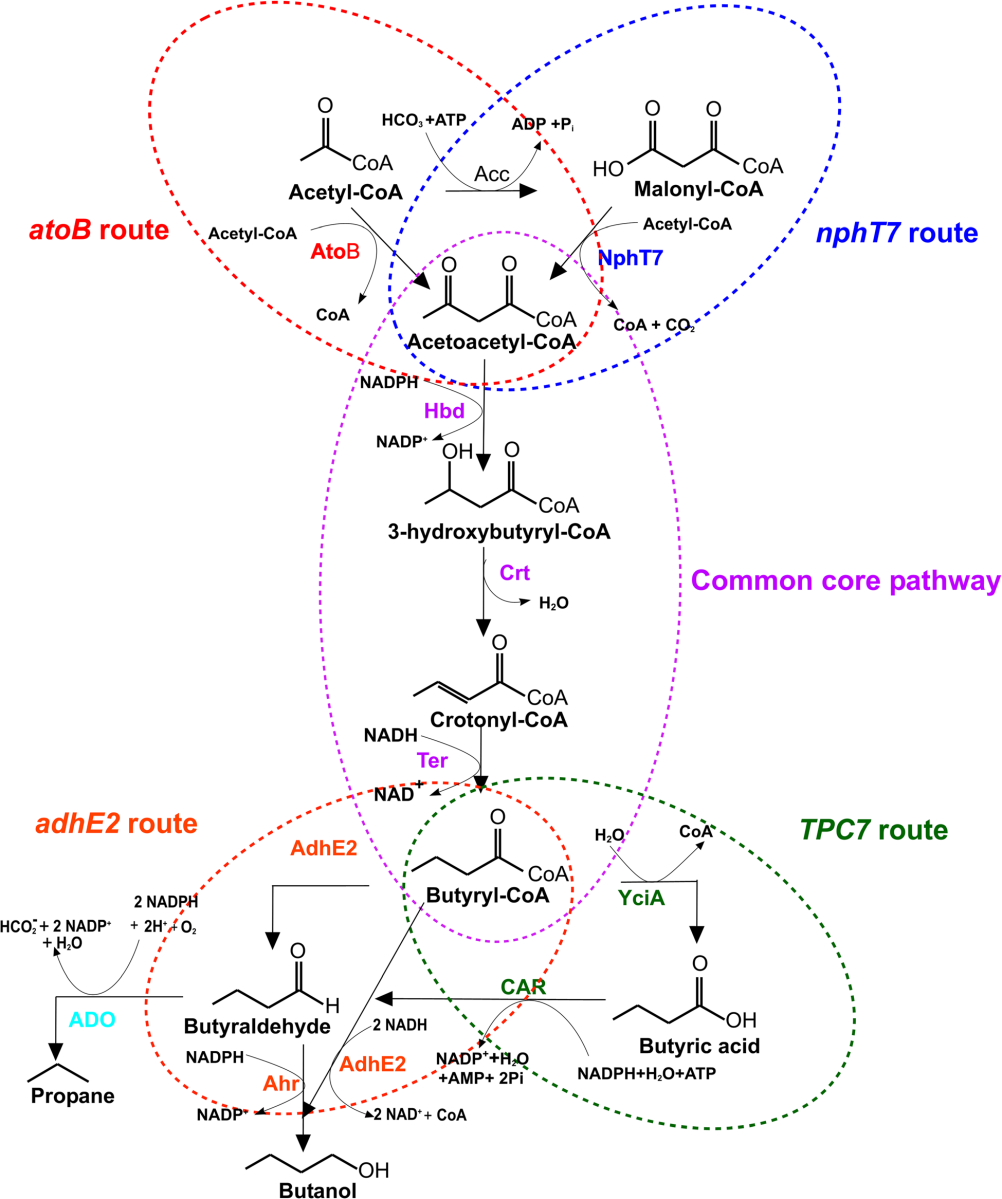
**The CoA-dependent butanol pathways used for the production of propane in** ***E. coli.*** The four CoA-dependent butanol producing synthetic routes (*atoB*-*adhE2* route, *atoB*-*TPC7* route, *nphT7*-*adhE2* route and *nphT7*-*TPC7* route) explored for butanol production in *E. coli* are shown. AdhE2, aldehyde-alcohol dehydrogenase; ADO, aldehyde deformylating oxygenase; Ahr, aldehyde reductase; AtoB, acetyl-CoA acetyltransferase; CAR, carboxylic acid reductase; Crt, 3-hydroxybutyryl-CoA dehydratase; Hbd, 3-hydroxybutyryl-CoA dehydrogenase; NphT7, acetoacetyl CoA synthase; Ter, trans-2-enoyl-CoA reductase; YciA, acyl-CoA thioester hydrolase.

Natural metabolic pathways for the renewable biosynthesis of propane do not exist. The discovery of an aldehyde deformylating oxygenase (ADO) from cyanobacteria, however, has paved the way for synthetic alkane pathways to be constructed [7-9]. A microbial platform for propane generation dependent on fatty acid biosynthesis was recently reported [10]. The authors concluded that the pathway was limited by total flux through fatty acid synthesis (FAS). The most obvious example of this limitation comes from the markedly enhanced rate of propane synthesis observed when fatty acids were supplied to the external media. In the present study, we sought to bypass this limitation by generating new synthetic pathways that are not dependent on FAS. In this study, we designed a series of modified butyraldehyde pathways based on the CoA-dependent butanol pathways commonly found in *Clostridium* spp. Propane biosynthesis was thereafter achieved by interrupting the route to alcohol by the addition of ADO (Figure 1).

The butanol pathway in *Clostridium* proceeds either via a keto acid route (Ehrlich pathway) or a CoA-dependent route. Higher yields of branched chain alcohols and aldehyde precursors (for example, isobutyraldehyde) from the decarboxylation of keto acids make the Ehrlich pathway less attractive because ADO has a strong preference for straight chain aldehyde substrates [11-13]. By contrast, butanol production by the CoA-dependent route initiates with the condensation of two molecules of acetyl CoA. Reduction in subsequent steps produces the end-product 1-butanol via a butyraldehyde intermediate. There are several reports of engineered CoA-dependent butanol pathways in *Escherichia coli* and other host organisms [14-20]. In the following work, we constructed and evaluated a series of CoA-dependent butyraldehyde pathways that eliminate the dependency on AdhE2, thereby allowing butyraldehyde to be re-routed towards propane instead of butanol.

The use of ADO (from *Prochlorococcus marinus* MIT9313) as a terminal decarbonylase has been used for the production of medium/long chain (C9-C17) as well as short chain-length alkanes (C3, C7) [8,10]. Variant forms of ADO have demonstrated improved activity with the shorter chain aldehydes that are not encountered in native cyanobacteria [7,12]. These variant forms of ADO are therefore attractive enzyme components for building new synthetic pathways with a greater productivity, as addressed in the current study.

Here, we report on the construction and evaluation of novel pathways for propane production in *E. coli* that are independent of the FAS pathway in a recent study, thereby opening up possibilities for further optimization of short chain-length alkane biosynthesis in future studies [10].

## Results

### Synthesis of butyraldehyde based on the CoA-dependent 1-butanol pathway

**Figure 2:**
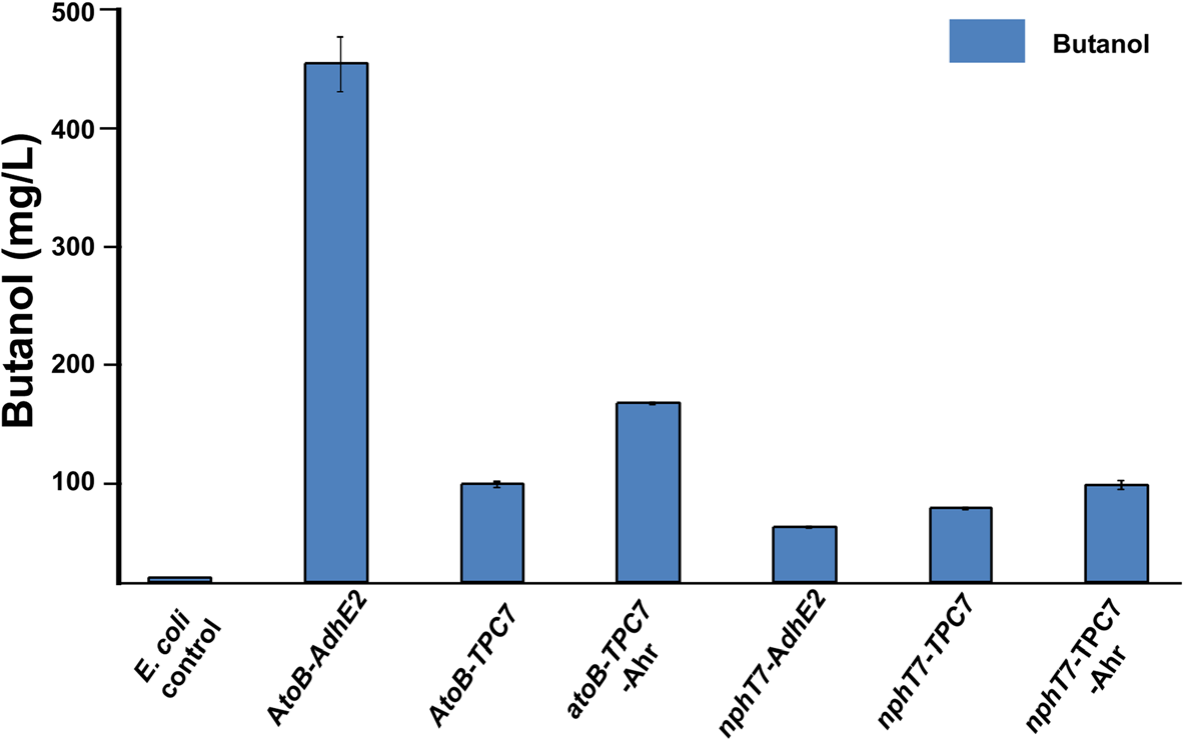
**Total butanol produced by the engineered** ***E. coli*** **BL21 strains** **.** Total butanol concentration obtained after 72 h of cultivation of *E. coli* wild-type cells harbouring engineered constructs is shown. The experimental protocol is described in the ‘Materials and methods’ section. Error bars are standard deviation (*n* = 4).

For propane production, the ADO enzyme requires butyraldehyde as a precursor. We therefore constructed two biosynthetic pathways for butyraldehyde synthesis based on the fermentation pathway of 1-butanol, as summarized in Figure 1. The initial step of the pathway consisted of i) either AtoB from *E. coli* (Figure 1; *atoB* route) or NphT7 from *Streptomyces* sp*.* (Figure 1; *nphT7* route) to convert the metabolic pathway intermediate, acetyl-CoA to acetoacetyl-CoA [17,21]. Although the latter step has not previously been evaluated in *E. coli*, it was recently shown to be superior to AtoB for butanol production in *Synechococcus elongatus* PCC 7942 [18]. For the second and third steps, clostridial 3-hydroxybutyryl-CoA dehydrogenase (Hbd) and 3-hydroxybutyryl-CoA dehydratase (crotonase or Crt) were used to convert acetoacetyl-CoA to crotonyl-CoA. For the fourth step, the oxygen-sensitive flavoenzyme butyryl-CoA dehydrogenase, present in the native clostridial pathway, was replaced with NADH-dependent trans-enoyl-CoA reductase (Ter) from *Treponema denticola* to reduce crotonyl-CoA to butyryl-CoA [21]. In the final step, butyraldehyde was synthesized by NAD(P)H-dependent reduction of butryl CoA catalysed by AdhE2. To verify pathway functionality for butyraldehyde synthesis, we used butanol as a molecular reporter since numerous studies have shown that it can be stably accumulated in *E. coli* and easily monitored [20]. Expression of all pathway components were confirmed by sodium dodecyl sulfate-polyacrylamide gel electrophoresis (SDS-PAGE) (Additional file 1: Figures S1 to S3), and pathway functionality was verified by mass spectrometry. Both routes led to the production of 1-butanol clearly indicating that both pathways were capable of butyraldehyde synthesis. The strain harbouring the *atoB* route led to a 6.2-fold higher production of 1-butanol production (473.3 ± 3.2 mg/L) compared to the nphT7 route (Figure 2).

### Modification of the butyraldehyde pathway by replacement of AdhE2

In the clostridial pathway, butyryl-CoA is converted to butanol in two successive catalytic steps by the bifunctional aldehyde/alcohol dehydrogenase AdhE2 (Figure 1; *AdhE2* route) [22]. From an engineering perspective, this is highly undesirable since the local presence of an aldehyde reductase component of AdhE2 is likely to compete for the ADO substrate. Given this possibility and to avoid creating internal metabolic competition, we therefore replaced AdhE2 with a i) thioesterase (YciA) from *Haemophilus influenzae* to cleave butyryl-CoA to butyric acid and ii) ATP/NADPH-dependent carboxylic acid reductase (CAR) from *Mycobacterium marinum* to convert butyric acid to butyraldehyde [8,23]. Two variant routes were thus created based on this modification, namely *atoB*-*TPC7*, *atoB*-*TPC7-ahr*, *nphT7*-*TPC7* and *nphT7*-*TPC7-ahr* (Figure 1; *TPC7* route). As before, 1-butanol was monitored to verify the functionality of the parts for butyraldehyde synthesis. In both cases, 1-butanol production was observed signifying yet again that butyraldehyde synthesis was achievable with both routes. 1-Butanol production was increased with the *atoB*-*TPC7* and *nphT7*-*TPC7* routes when Ahr was overexpressed (1.5 and 1.3 times for *atoB*-*TPC7* and *nphT7*-*TPC7* routes, respectively), indicating that the conversion of butraldehyde to butanol by the endogenous aldehyde reductases was limiting total pathway flux.

### Evaluation of pathway routes for propane synthesis

**Figure 3:**
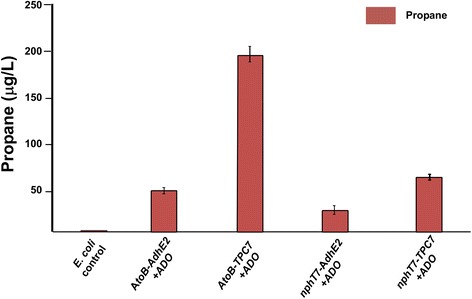
**Total propane produced by the engineered** ***E. coli*** **BL21 strains** **.** Total propane accumulated over 4 h of reaction under assay conditions performed in GC vials for the pathway-engineered *E. coli* cells, after overexpressing with ADO. The experimental protocol is described in the ‘Materials and methods’ section. Error bars are standard deviation (*n* = 4). ADO, aldehyde deformylating oxygenase; IPTG, isopropyl β-D-1-thiogalactopyranoside.

The combined analysis clearly indicated that all pathway components for butyraldehyde synthesis were functional. In order to convert butyraldehyde to propane, the aldehyde reductases in the four butanol pathways were thereafter replaced or complemented by ADO. The TPC7-based strains produced relatively more propane (*atoB-TPC7-ADO* and *nphT7-TPC7-ADO*) compared to other strains (Figure 3; Additional file 1: Figures S4 and S5), and the majority of the pathway flux in all four routes was still directed towards 1-butanol. Interestingly, the lack of propane with the two *AdhE2* pathways clearly supported our initial hypothesis regarding internal metabolic competition with AdhE2.

**Figure 4:**
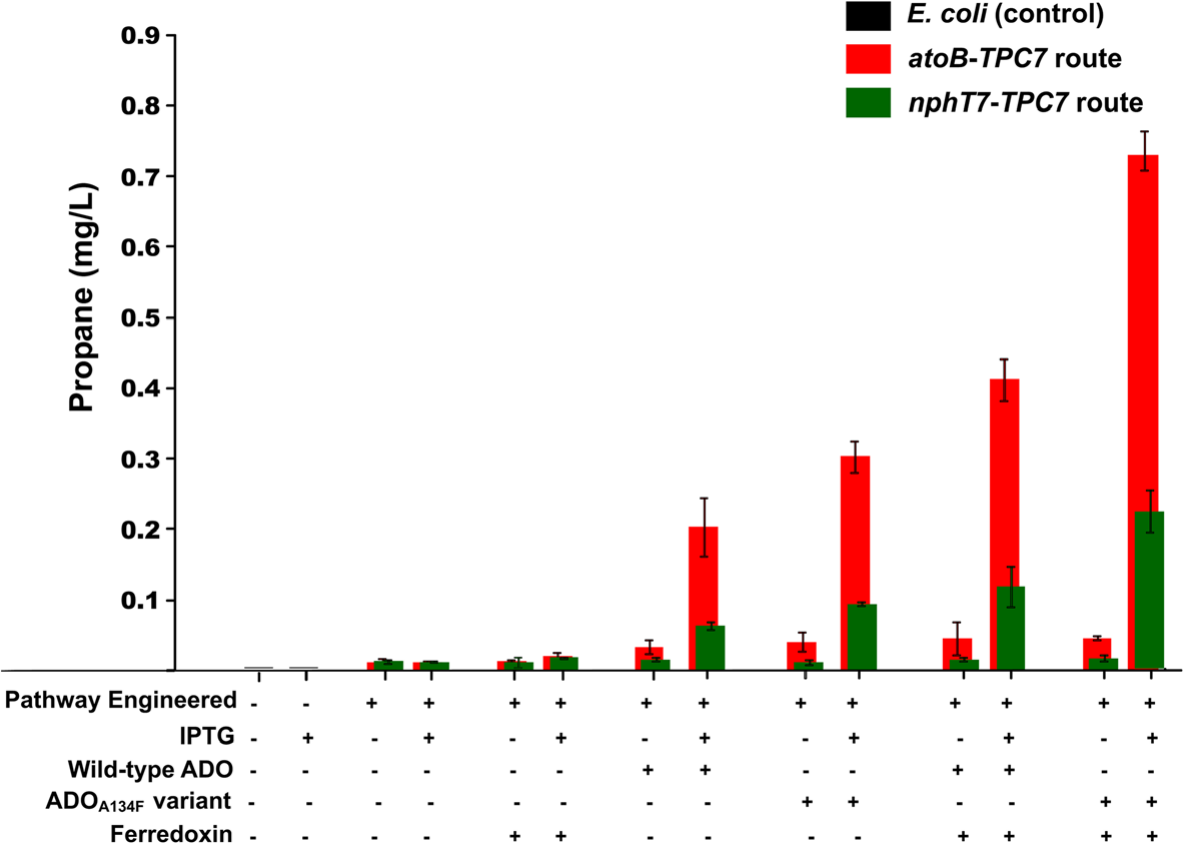
**Propane production in pathway-engineered** ***E. coli*** **strains containing wild-type ADO or the ADO**_**A134F**_ **variant enzyme.** *E. coli* cells were either pathway-engineered to include the *atoB*-*TPC7* route (indicated by red colour) or the nphT7-*TPC7* route (indicated by green colour) and contain wither wild-type ADO or the ADO_A134F_ variant enzyme in the absence/presence of ferredoxin (Fdx) from *Synechocystis* sp. PCC 6803. A detailed protocol for the pathway engineering and propane detection is included in the ‘Materials and methods’ section and with the supporting information (Additional file 1: Figure S6). Error bars are standard deviation (*n* = 4).

The two functional *TPC7*-dependent propane pathways were subsequently modified by replacing ADO with the ADO_A134F_ variant enzyme (Figure 4). This enzyme was initially designed to overcome some of the kinetic constraints in propane production resulting from very low activity of the enzyme towards short-chain substrates [12]. The A134F substitution alters the topology of the substrate-access channel and has been shown to result in improved activity towards low chain-length aldehydes butyraldehyde and pentaldehyde. The ADO_A134F_ has also been shown to generate propane in both *in vitro* and *in vivo* biotransformations, and the *E. coli* strain expressing the variant enzyme produced approximately two times more propane (0.47 ± 0.04 mg/L) in comparison to the corresponding wild-type ADO system when fed with externally added butyraldehyde [12]. As expected, introduction of the variant ADO into the pathways engineered in this study also resulted in enhanced propane production in all cases (Figure 4). The best result was obtained with the *atoB-TPC7*-*ADO*_*A134F*_ combination, showing a 1.8-fold improvement, in comparison to the wild-type ADO system, and reaching a maximum propane titre of 0.29 ± 0.02 mg/L (Figure 4).

### Increasing electron supply to ADO via ferredoxin

It has previously been shown that the supply of electrons to ADO via endogenous enzymes was inadequate for effective alkane biosynthesis in *E. coli* [10]. To alleviate this constraint, the heterologous ferredoxin PetF (*ssl0020*), the presumed natural electron acceptor/donor to ADO in *Synechocystis* sp*.* PCC 6803, was overexpressed as part of the engineered pathways. Consequently, co-expression of PetF improved propane production by roughly twofold for both the *atoB-TPC7* and *nphT7-TPC7* pathway in combination with both ADO and ADO_A134F_ (Figure 4). In order to verify the impact of the optimization efforts, residual butanol was also measured in selected strains (Additional file 1: Figures S4 and S5). If ADO was able to compete for butyraldehyde, the common precursor for both propane and butanol, one would expect to observe a lower butanol titre in strains with enhanced propane production. Indeed, residual butanol levels were decreased when ADO and ferredoxin were overexpressed. Similarly, when the ADO_A134F_ variant replaced wild-type ADO, the accumulation of butanol in the media was also lower. This is consistent with the terminal ADO (and the ADO_A134F_ variant) acting on butyraldehyde produced by the engineered synthetic pathway (Additional file 1: Figures S4 and S5).

### Removing competing pathways for propane synthesis

**Figure 5:**
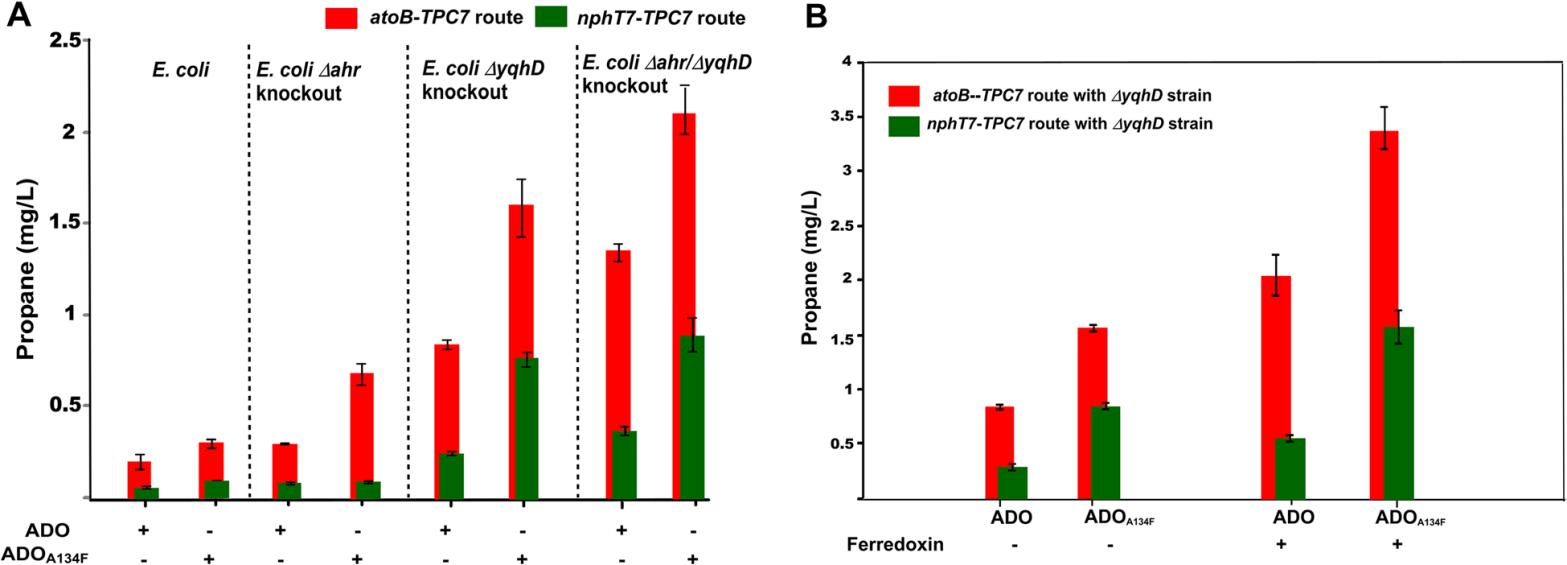
**Propane produced in pathway-engineered Δahr/ΔyqhD single or double knockout** ***E. coli*** **strains and the effects of co-expressing a ferredoxin electron donating system.** Propane production in the pathway-engineered *ΔyqhD* knockout cells with wild-type ADO or with the ADO_A134F_ variant enzyme is shown **(A)**. The *ΔyqhD* knockout strains were either engineered to contain the *atoB*-*TPC7* route (indicated by red colour) or the *nphT7-TPC7* route (indicated by green colour). Wild-type ADO or the ADO_A134F_ variant enzyme was co-expressed in the engineered cells either in combination with or without ferredoxin (Fdx) from *Synechocystis* sp. PCC 6803 **(B)**. A detailed protocol for the pathway engineering and propane detection is included in the ‘Materials and methods’ section and supporting information (Additional file 1: Figure S6). Error bars are standard deviation (*n* = 4). ADO, aldehyde deformylating oxygenase.

*E. coli* cells contain a wide range of aldehyde reductases and alcohol dehydrogenases which act to scavenge potentially toxic intracellular aldehydes [24]. Two of the native aldehyde reductases in *E. coli*, Ahr and YqhD, were previously shown to compete for butyraldehyde [10]. In order to further optimize propane production, *Δahr* and *ΔyqhD* single and double knockout *E. coli* strains were therefore tested (Figure 5A). Among the single knockout strains, *ΔyqhD* showed a greater increase in propane production compared to *Δahr*. The double knockout strain *Δahr/ΔyqhD* showed a cumulative effect of the gene knockouts, reaching a propane titre of 2.05 ± 0.12 mg/L for the *atoB-TPC7*-*ADO*_*A134F*_ strain. The single knockout strains were also tested for propane production in the presence of *Synechocystis* sp. ferredoxin (Fdx), reaching a titre of up to 3.40 ± 0.19 mg/L in the *ΔyqhD* background (Figure 5B).

### Potential for large-scale production of propane

**Figure 6:**
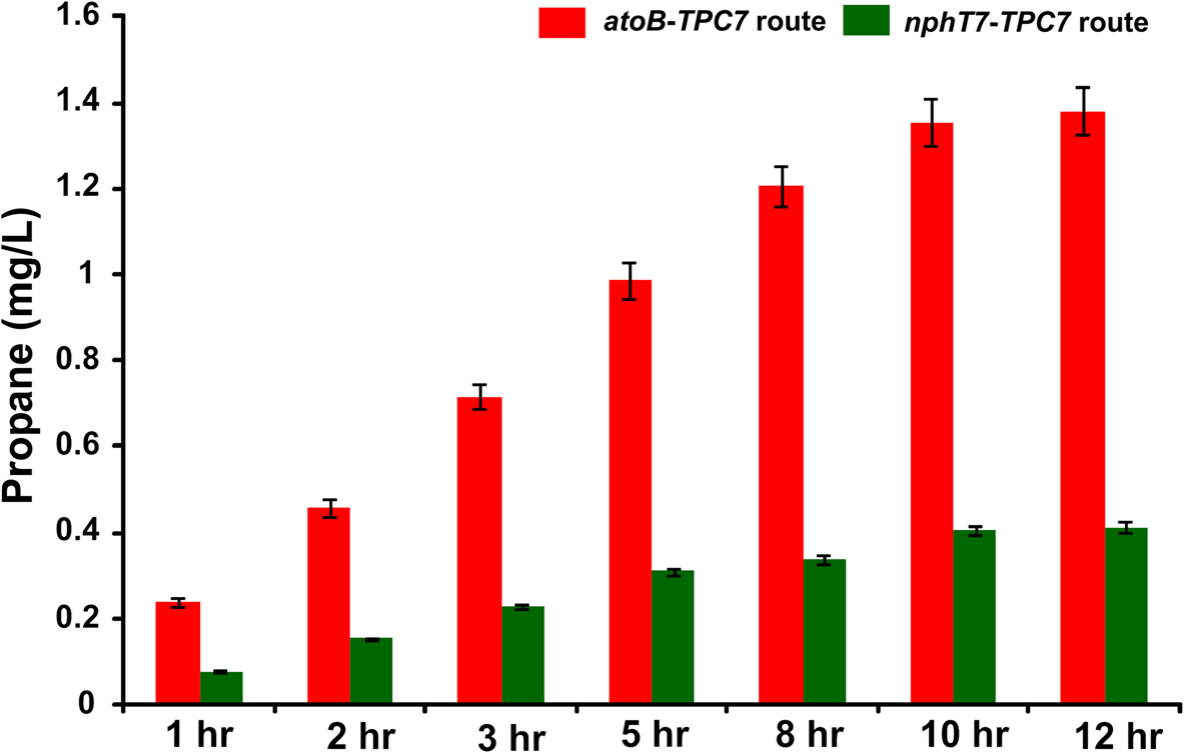
**Larger scale cultures of the best performing propane-producing pathways** **.** The *atoB*-*TPC7* route (indicated by red colour) and *nphT7*-*TPC7* (indicated by green colour) engineered in *ΔyqhD* knockout cells in the presence of the ADO_A134F_ variant and ferredoxin system were analysed at larger scale. The culture volume was scaled up to 400-fold to 200 mL, in a 300-mL flask sealed with airtight rubber septum. The propane accumulation for 12 h is shown. Error bars are standard deviation (*n* = 3).

As propane biosynthesis was only analysed in small-scale cultures (2.0 mL gas chromatography (GC) vials), larger scale productivity and longevity of propane production was also investigated with the best performing strains (that is, *atoB*-*TPC7* or *nphT7*-*TPC7* routes incorporated into *E. coli ΔyqhD* in the presence of the ADO_A134F_ variant and ferredoxin). The culture volume was scaled 400-fold to 200 mL, in 300-mL flasks sealed with airtight rubber septa, and propane production and accumulated residual butanol were monitored over 12 h (Figure 6 and Additional file 1: Figure S7). The highest propane accumulation was observed between 8 and 10 h. For the *atoB*-*TPC7* route, 1.38 ± 0.06 mg/L propane was accumulated within 12 h, while 0.4 ± 0.1 mg/L propane was accumulated for the *nphT7*-*TPC7* pathway (Figure 6). The ability to scale propane production is an important proof-of-concept that suggests large-scale propane production should be possible in a fermentor setup with appropriate optimisation of growth and feeding conditions. Controlled cultivation in a fermentor should bypass potential problems associated with flask cultivation, for example, non-optimal oxygen supply and the production of undesirable byproducts.

## Discussion

Recent progress in exploring various heterologous microbial platforms towards metabolites such as fatty acids, alcohols and alkanes has improved the prospect of advanced biofuel production. Propane (C_3_H_8_) was proposed as a new microbial biofuel target as it would be able to act as a direct drop-in replacement for the corresponding non-renewable products currently in use (for example, autogas, LPG). Propane also has very good physicochemical properties which allow it to be stored and transported in a compressed liquid form, while under ambient conditions, it is a clean-burning gas used in various applications ranging from heating to utilization as a transport fuel. The very first microbial biosynthetic pathway for producing propane was recently engineered in *E. coli* [10]*.* In that study, it was found that the biosynthesis of propane was restricted by the poor activity of the enzyme ADO towards short-chain substrates in combination with competing pathways that limits the availability of the ADO substrate, butyraldehyde. The study reported here therefore explored alternative biosynthetic approaches to provide a more comprehensive understanding of the limiting factors in microbial propane biosynthesis and to find possible ways to overcome metabolic bottlenecks.

The pathways assembled in the present study are not dependent on type II fatty acid biosynthesis unlike in the previous study and instead use CoA- rather than ACP-based intermediates. Importantly, this strategy allowed us to bypass the strict regulatory control on native fatty acid biosynthesis flux [10,25,26]. The pathways engineered in this study comprise four parallel variations of the Clostridial butanol pathway, differing from one another in the conversion of (1) acetyl-CoA to acetoacetyl-CoA (*atoB vs. nphT7* routes) and (2) butyryl-CoA to butyraldehyde (*adhE2 vs. TPC7* routes). In the first stage of pathway evaluation, production of butanol was used as a measure of metabolic flux towards butyraldehyde, the immediate substrate for propane production by ADO. The TPC7 variant routes were developed in order to avoid the bifunctional activity of AdhE2 which includes the reduction of butyraldehyde to 1-butanol. Such a reaction would be expected to compete with ADO for the butyraldehyde intermediate and lower propane production. Therefore, in order to separate these functional activities and allow greater pathway flexibility, AdhE2 was replaced with a ‘TPC7’ module in combination with Ahr.

For the first two variant pathways, the (co)substrate requirements for AtoB and NphT7 are quite distinct; AtoB requires two molecules of acetyl CoA whereas nphT7 requires acetyl-CoA along with malonyl-CoA. Both reactions result in the release of CoA, though in the latter case, there is also release of CO_2_. The greater 1-butanol levels observed with the AtoB pathway suggest that it is a preferred route for acetoacetyl CoA production in *E. coli*. However, it is also important to note that AtoB has a higher protein expression level in *E. coli* compared to nphT7 (Additional file 1: Figure S1), and this may have limited 1-butanol production via the *nphT7* route. When coupled with ADO, butyraldehyde is converted to propane instead of butanol, and propane production was observed in the majority of the alternative strains. Consequently, the most productive propane pathway in the wild-type *E. coli* cells was observed with the *atoB-TPC7-ADO* route (220 ± 3 μg/L). The flux towards propane through these pathways was compromised by competing native aldehyde reductase activites, as demonstrated by a significant increase in propane production and a concomitant reduction in butanol production following the deletion of two native genes encoding for such activities. In contrast to the two *TPC7* pathways, the *adhE2*-dependent routes produced significantly less propane. This observation reinforced the view that AdhE2 converts butyraldehyde to butanol in successive steps without release of the butyraldehyde, thus preventing efficient interception of the pathway by ADO towards propane.

Quantitative comparisons revealed a difference of almost 3 orders of magnitude between the titres of butanol and propane (173 *vs*. 0.2 mg/L) in the wild-type strains of AtoB-TPC7-Ahr and AtoB-TPC7-ADO. This difference can be attributed partly to the poor efficiency of the last biosynthetic step (conversion of butyraldehyde to propane). This is consistent with the earlier reports that have demonstrated *in vitro* and *in vivo* in *E. coli* that ADO has very low activity even towards the most preferred native substrates (C10-C15) and especially towards C4 substrates [10,12]. To partially alleviate this constraint, the ADO_A143F_ variant was introduced into the engineered pathways to replace native ADO. This resulted in a 1.8-fold improvement in propane, most likely reflecting more efficient utilization of the intracellular pool of available butyraldehyde. However, the remaining large difference between 1-butanol and propane levels suggests that the optimization efforts employed in the present study are still not sufficient and/or that other metabolic constraints are limiting. One obvious example, as confirmed in this study as well as others, is the reduction of ADO in the presence of a 2Fe-2S ferredoxin [27]. Ferredoxin systems are known to improve the metabolic reactions by mediating electron transfer in partner enzymes as observed here [27-29]. Such an approach could be further aided by additional reducing components such as NAD(P)H:flavodoxin/ferredoxin oxidoreductase (for example, fpr) as demonstrated in the previous work [10].

The present work introduces an alternative and conceptually different pathway for propane production in comparison to the previous work [10] despite the fact that the propane titre remained slightly lower. However, a direct comparison between the FAS and CoA pathways is not suitable, particularly since the analysed experimental parameters differed between the two studies. Both the current, and previously reported, pathways will benefit from further optimisation strategies to improve carbon flux, involving detailed targeted metabolomics and mass balance calculation, pathway modelling and pathway refactoring along with modifications to the known limitations in the turnover rate constant of ADO.

## Conclusions

We have reported here new pathways for propane production based on a fermentative butanol pathway. These are distinct from previously reported pathways that were based on fatty acid synthesis, where propane production is limited by the availability of butyraldehyde precursors and the poor activity of ADO with butyraldehyde. We have demonstrated that the new pathways are plausible alternatives for the construction of next-generation microbial propane production platforms. While the majority of the metabolic flux is still directed towards other products (that is, butanol), our work also demonstrates that there is strong potential for further optimization aimed at increasing propane production.

## Materials and methods

All chemicals, solvents and standards were purchased from Sigma-Aldrich (St. Louis, MO, USA) and Fisher Scientific (Waltham, MA, USA) and were of analytical grade. Media components were obtained from Formedium (Norfolk, UK). Gene sequencing and oligonucleotide synthesis were performed by Eurofins MWG (Ebersberg, Germany). D-Glucose (GOPOD Format) assay kit was from Megazyme (Wicklow, Ireland).

### Strains and plasmids

**Figure 7:**
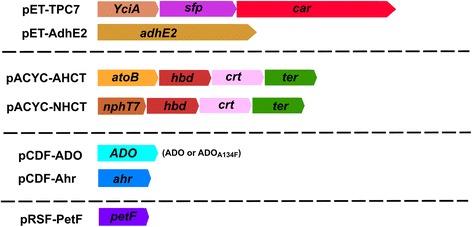
**The plasmid design used to construct engineered propane producing pathways in** ***E*** *.* ***coli.*** The structure of all plasmids used in this study for *E. coli* pathway engineering is shown. The preparation and design of these plasmids are described in the ‘Materials and method’ section.

BL21 (DE3) (*fhuA2 [lon] ompT gal (λ DE3) [dcm] ΔhsdS*) cells from Novagen (Madison, WI, USA) were used for protein expression. The *ahr* (GenBank ID: ACT44688.1) and *yqhD* (GenBank ID: AAA97166.1) single and double knockout strains were generated in a previous study [10]. The structure of all plasmids is graphically depicted in Figure 7, and their preparation is described below.

### pET-TPC7

The pET-TPC7 plasmid was constructed in a pETDuetT-1 vector (ColE1 replicon, Ampicillin^R^, from Novagen) by replacing TE4 acyl-ACP thioesterase with a synthesized gene encoding YciA (*Haemophilus influenza*, GenBank ID: AAC22485.1) in pET-TPC4 backbone plasmid described in the previous study using restriction sites HindIII and NcoI [10]. The final plasmid contained synthesized genes encoding Sfp (maturation factor phosphopantetheinyl transferase from *Bacillus subtilis*, GenBank ID: X65610.1) and CAR (carboxylic acid reductase from *M. marinum*, GenBank ID: ACC40567.1) upstream to *yciA*.

### pACYC-NHCT

The pACYC-NHCT plasmid was constructed in a pACYCDuet-1 vector (P15A replicon, Chloramphenicol^R^, from Novagen) by subcloning a synthesized NcoI-BamHI gene fragment (GenScript, Piscataway, NJ, USA) carrying genes encoding NphT7 (acetoacetyl-CoA synthase from *Streptomyces* sp*.* CL190, GenBank ID: D7URV0.1) and Hbd (3-hydroxybutyryl-CoA dehydrogenase from *Clostridium acetobutylicum* ATCC 824, GenBank ID: P52041.2) into a synthesized pACYC construct harbouring *crt* (3-hydroxybutyryl-CoA dehydratase from *C. acetobutylicum* ATCC 824, GenBank ID: P52046.1) and *ter* (trans-2-enoyl-CoA reductase from *T. denticola* ATCC 35405, GenBank ID: Q73Q47.1).

### pACYC-AHCT

pACYC-AHCT is a pACYCDuet-1 vector (P15A replicon, Chloramphenicol^R^, from Novagen) with *atoB* (Acetyl-CoA acetyltransferase from *E. coli*, GenBank ID: P76461.1), *hbd* (3-hydroxybutyryl-CoA dehydrogenase from *C. acetobutylicum* ATCC 824, GenBank ID: P52041.2), *crt* (3-hydroxybutyryl-CoA dehydratase from *C. acetobutylicum* ATCC 824, GenBank ID: P52046.1) and *ter* (trans-2-enoyl-CoA reductase from *T. denticola* ATCC 35405, GenBank ID: Q73Q47.1) genes inserted in the order. Gene *atoB* (acetyl-CoA acetyltransferase from *E. coli*, GenBank ID: P76461.1) was PCR-amplified from *E. coli* K-12 purified genome, using primers 5′-attaggtaccAAAAATTGTGTCATCGTCAGTGCGGTAC and 5′-attaaagcTTAATTCAACCGTTCAATCACCATCGCAAT, showing complementary regions in capital letters. The *atoB* PCR fragment was then subcloned into pACYC-NHCT, thus replacing the KpnI-HindIII fragment carrying NphT7, resulting pACYC-AHCT plasmid.

### pET-AdhE2

The pET-AdhE2 plasmid was constructed in a pET-Duet vector (f1 origin, Ampicillin^R^, from Novagen) with *adhE2* by subcloning synthesized gene encoding AdhE2 (aldehyde-alcohol dehydrogenase, from *C. acetobutylicum* ATCC 824, GenBank ID: Q9ANR5) from a pUC57 parent vector provided by GenScript into pET-Duet vector, using restriction sites NcoI and AvrII.

### pCDF-ADO and pCDF-ADO_A134F_

The pCDF-ADO plasmid was constructed in a pCDFDuet-1 vector (CDF replicon, streptomycin/spectinomycin^R^, from Novagen) with ADO (aldehyde deformylating oxygenase from *Prochlorococcus marinus* MIT9313, GenBank ID: Q7V6D4.1) cloned into the vector using NcoI and EcoRI restriction sites. The pCDF-ADO_A134F_ vector was created by mutating the ADO insert, using A134F_forward (5′-GCA TTT GCG ATT TCT *TTT* TAT CAT ACG TAC-3′) and A134F_reverse primers (5′-GTA CGT ATG ATA *AAA* AGA AAT CGC AAA TGC-3′). The correct mutations were confirmed by complete plasmid DNA sequencing. Gene encoding ADO was originally provided by E. Neil G. Marsh (Department of Biological Chemistry, University of Michigan, USA) in a pET28b-cAD vector which was used for the previous study [30].

### pCDF-Ahr

The pCDF-Ahr plasmid was constructed in a pCDFDuet-1 vector (CDF replicon, streptomycin/spectinomycin^R^, from Novagen). Gene encoding Ahr (aldehyde reductase from *E. coli* GenBank ID: P27250.2) was PCR-amplified from isolated *E. coli* K-12 genomic DNA, using primers 5′ATTAATCCATGGTCTAGATAATTAATGGATCCAGGAGGAAACATATGTCGATGATAAAAAGCTATGCCGCAAAAG-3′ and 5′-ATTAATCCTAGGAAGCTTCTCGAGTCAAAAATCGGCTTTCAACACCACGCGG-3′, and cloned into using restriction sites NcoI and AvrII [8].

### pRSF-PetF

The pRSF-PetF plasmid was constructed in a pRSF-Duet1 vector (RSF replicon, Kanamycin^R^, from Novagen) with *fdx* (ferredoxin from *Synechocystis* sp. PCC 6803, GenBank ID: WP_010873424.1) by subcloning synthesized gene from a pUC57 parent vector provided by GenScript into a pRSF-Duet1 vector using NcoI and AvrII restriction sites.

### Co-expression and introduction of the pathway in *E. coli*

The *atoB*-*adhE2* route was introduced into *E. coli* or the knockout cells by co-expressing pACYC-AHCT and pET-AdhE2 vectors. The *atoB*-*TPC7* route was introduced into *E. coli* or the knockout cells by co-expressing pACYC-AHCT and pET-TPC7 vectors. The *nphT7*-*adhE2* route was introduced into *E. coli* or the knockout cells by co-expressing pACYC-NHCT and pET-AdhE2 vectors. The *nphT7*-*TPC7* route was introduced into *E. coli* or the knockout cells by co-expressing pACYC-NHCT and pET-TPC7 vectors in the cells.

*E. coli* cells containing engineered pathways were further engineered by co-transforming either pCDF-ADO or pCDF-ADO_A134F_ vectors in order to introduce ADO or the ADO_A134F_ variant. The pRSF-PetF vector was co-expressed for Fdx, while pCDF-Ahr was used to introduce Ahr enzyme in the pathway. The presence of all the proteins in each individual plasmid was confirmed by SDS-PAGE and mass spectrometry analysis of the respective SDS-PAGE bands. In the case of proteins with a hexahistidine tag, Western blotting (using WesternBreeze Chemiluminescent Immunodetection kit from Invitrogen, Carlsbad, CA, USA) was also used to analyse the expression of his-tagged proteins.

### SDS-PAGE analysis of protein expression in cells containing plasmids encoding pathway components

Protein expression levels in cells containing plasmids that encode the pathway enzymes were examined by SDS-PAGE. T5 media (20 mL; 12 g tryptone, 24 g yeast extract, 4 mL glycerol, 12.5 g K_2_HPO_4_, 2.3 g KH_2_PO_4_, 20 g glucose per litre) was inoculated with 1% (*v*/*v*) transformed *E. coli* cells and incubated at 37°C (180 rpm) until the optical density at 600 nm (OD_600nm_) reached 0.5. Cultures were then induced with isopropyl β-D-1-thiogalactopyranoside (final concentration of 0.5 mM). Cultures were grown for a further 24 h at 30°C (180 rpm). Samples (200 μL) from the cultures were taken for SDS PAGE analysis. Samples were taken before isopropyl β-D-1-thiogalactopyranoside (IPTG) induction and after 4 or 24 h of IPTG induction and cells harvested by centrifugation. Samples were electrophoresed in 12% RunBlue precast SDS-PAGE gels (Expedeon, Cambridge, UK). Protein bands were visualized by staining with Instant Blue protein stain (Expedeon).

### Media, cultivation and detection of propane and butanol

Lysogeny broth (LB) liquid media (10 mL) was inoculated using *E. coli* glycerol stocks (from −80°C) and incubated at 37°C overnight at 180 rpm. Of T5 media, 50 mL (12 g tryptone, 24 g yeast extract, 4 mL glycerol, 12.5 g K_2_HPO_4_, 2.3 g KH_2_PO_4_, 20 g glucose per litre) was inoculated with 1% (*v*/*v*) of the inoculum and kept for incubation at 37°C (180 rpm) until the optical density at 600 nm (OD_600_) reached 0.5. The cultures were then induced with IPTG to a final concentration of 0.5 mM. The cell cultures were further grown for 4 h at 30°C and 180 rpm to prepare the samples for propane detection. In the case of total butanol produced in the butanol pathway, the culture was left at 30°C (180 rpm) for 72 h, while for residual butanol detection, samples were taken from this culture when OD_600_ reached 1.5. Control cultures were made using untransformed *E. coli* strain BL21 DE3 cells.

For propane formation analysis, 50 mL cell culture was centrifuged at 4,000 rpm, and the supernatant was discarded. The cell pellets were resuspended in a 6.25-mL T5 media with 0.5 mM IPTG, and 500 μL of the resuspended culture was transferred into 2-mL crimp sealed GC vial and used for propane analysis. The vials were incubated at 30°C, with shaking at 180 rpm for 3 h. Headspace of 1.0 mL from the cultures grown in the GC vial was manually removed and injected into the GC with a gas tight syringe. Propane detection was carried out using a Varian 3800 GC (Palo Alto, CA, USA) equipped with a DB-WAX column (30 m × 0.32 mm × 0.25 μM film thickness, J&W Scientific, Santa Clara, MA, USA). The injector temperature was 250°C with a split ratio of 10:1. The column temperature was set from 40°C hold for 2 min to 100°C at 20°C/min with helium flow at 1 mL/min and FID temperature at 250°C. Propane peak was identified by comparing it with the analytical propane standard, and quantification was done using a propane calibration curve.

For residual butanol detection, 50 mL liquid culture was spun down at 4,000 rpm for 10 min. Of the supernatant, 500 μL was extracted with 500 μL of ethyl acetate containing 0.2% hexane as internal standard and dried over MgSO_4_. Of the sample, 1 μL was analysed in GC using a Varian 3800 GC equipped with a HP-5 column (30 m × 0.32 mm × 0.25 μM film thickness, JW Scientific). The injector temperature was 250°C with a split ratio of 20:1. The column temperature was set from 40°C hold for 1 min to 280°C at 20°C/min with helium flow at 1 mL/min and FID temperature at 250°C. Butanol peak was identified by comparing with the analytical butanol standard, and quantification was done using a butanol calibration curve.

## Additional file

Additional file 1:
**Additional information. Figure S1.** *SDS-PAGE analysis of overexpressed proteins in E. coli cells transformed with plasmids pACYC-AHCT, pACYC-NHCT and pCDF-Ahr.* The SDS-PAGE gel shows IPTG uninduced and induced *E. coli* cells transformed with pACYC-AHCT, pACYC-NHCT and pCDF-Ahr vectors (sections I, II and III, respectively). Lane 1 is the standard protein marker and in each section, lane 2 shows uninduced cells, lane 3 shows IPTG induced cells after 4 h and lane 4 is the IPTG induced cells after 24 h. AtoB (40.5 kDa); NphT7,(34.6 kDa); Hbd (30.6 kDa); Crt (28.2 kDa); Ter (43.8 kDa) and Ahr (37.8 kDa) are marked. **Figure S2.** *SDS-PAGE analysis of overexpressed proteins in E. coli cells transformed with pET-adhE2 and pET-TPC7 vectors.* The SDS-PAGE gel shows IPTG uninduced and induced *E. coli* cells transformed with pET-AdhE2 and pET-TPC7 vectors (shown in sections I and II, respectively). Lane 1 is the standard protein marker and in each section, lane 2 shows uninduced cells and lane 3 is the IPTG induced cells after 24 h. The band positions of AdhE2 (94.4 kDa); CAR (128.9 kDa); sfp (26.2 kDa) and YciA (16.7 kDa) are shown. **Figure S3.** *SDS-PAGE analysis of overexpressed proteins in E. coli cells transformed with pCDF-ADO and pRSF-PetF vectors.* The SDS-PAGE gel shows IPTG uninduced and induced *E. coli* cells transformed with pCDF-ADO and pRSF-PetF vectors (shown in sections I and II respectively). Lane 1 is the standard protein marker and in each section, lane 2 shows uninduced cells, and lane 3 is the IPTG induced cells after 24 h. The positions of ADO (29.3 kDa) and *Fdx*, ferredoxin (10.4 kDa) are shown. **Figure S4.** *Residual butanol produced in E. coli and knockout strains engineered to harbour the atoB*-*TPC7 pathway.* Residual butanol produced in the pathway engineered *E. coli* or knockout strains with wild-type ADO or with the ADO_A134F_ variant enzyme are shown. *E. coli and Δahr/ΔyqhD* single or double knockout strains were engineered to contain the *atoB*-*TPC7* route (indicated by red colour). The effect of wild-type ADO or the ADO_A134F_ variant enzyme with or without *Synechocystis* ferredoxin (Fdx) was also analysed. **Figure S5.** *Residual butanol produced in E. coli and knockout strains engineered to harbour the nphT7*-*TPC7 pathway*. Residual butanol produced in the pathway engineered *E. coli* or knockout strains with wild-type ADO or with the ADO_A134F_ variant enzyme are shown. *E. coli and Δahr /ΔyqhD* single or double knockout strains were engineered to contain the *nphT7*-*TPC7* route (indicated by green colour). The effect of wild-type ADO or the ADO_A134F_ variant enzyme with or without *Synechocystis* ferredoxin (Fdx) was also analysed. **Figure S6.** *GC traces and the corresponding propane peak integrated for pathway analysis*. GC traces showing the integrated propane peak for the *AtoB-TPC7* route containing *E. coli BL21* strains coexpressed with ADO and ferredoxin (trace a), *AtoB-TPC7* route containing *E. coli BL21* strains coexpressed with ADO (trace b), *AtoB-TPC7* route engineered in *E. coli BL21* strains coexpressed with ferredoxin in absence of ADO (trace c), *AtoB-TPC7* route engineered in *E. coli BL21* strains (trace d) and control *E. coli BL21* cells without pathway engineering (trace e) are shown. **Figure S7.** *Time course for residual butanol accumulation in* large-scale cultures of the best propane-producing pathways. The *atoB*-*TPC7* route (indicated by red symbols) and *nphT7*-*TPC7* (indicated by green symbols) engineered in *ΔyqhD* knockout cells in the presence of the ADO_A134F_ variant and ferredoxin system were analysed at larger scale.

## Abbreviations

AdhE2: aldehyde-alcohol dehydrogenase
ADO: aldehyde deformylating oxygenase
ahr: aldehyde reductase
CAR: carboxylic acid reductase
FAS: fatty acid synthesis
Fdx: ferredoxin
GC: gas chromatography
IPTG: isopropyl β-D-1-thiogalactopyranoside
OD_600_: optical density at 600 nm
Ter: trans-2-enoyl-CoA reductase
