# Supplementary material for: A microbial platform for renewable propane synthesis based on a fermentative butanol pathway
Source: Biotechnol Biofuels. 2015 Apr 10;8:61. doi: 10.1186/s13068-015-0231-1 (PMC4392459; doi:10.1186/s13068-015-0231-1)
Supplement: Additional file 1: — Additional information. Figure S1. SDS-PAGE analysis of overexpressed proteins in E. coli cells transformed with plasmids pACYC-AHCT, pACYC-NHCT and pCDF-Ahr. The SDS-PAGE gel shows IPTG uninduced and induced E. coli cells transformed with pACYC-AHCT, pACYC-NHCT and pCDF-Ahr vectors (sections I, II and III, respectively). Lane 1 is the standard protein marker and in each section, lane 2 shows uninduced cells, lane 3 shows IPTG induced cells after 4 h and lane 4 is the IPTG induced cells after 24 h. AtoB (40.5 kDa); NphT7,(34.6 kDa); Hbd (30.6 kDa); Crt (28.2 kDa); Ter (43.8 kDa) and Ahr (37.8 kDa) are marked. Figure S2. SDS-PAGE analysis of overexpressed proteins in E. coli cells transformed with pET-adhE2 and pET-TPC7 vectors. The SDS-PAGE gel shows IPTG uninduced and induced E. coli cells transformed with pET-AdhE2 and pET-TPC7 vectors (shown in sections I and II, respectively). Lane 1 is the standard protein marker and in each section, lane 2 shows uninduced cells and lane 3 is the IPTG induced cells after 24 h. The band positions of AdhE2 (94.4 kDa); CAR (128.9 kDa); sfp (26.2 kDa) and YciA (16.7 kDa) are shown. Figure S3. SDS-PAGE analysis of overexpressed proteins in E. coli cells transformed with pCDF-ADO and pRSF-PetF vectors. The SDS-PAGE gel shows IPTG uninduced and induced E. coli cells transformed with pCDF-ADO and pRSF-PetF vectors (shown in sections I and II respectively). Lane 1 is the standard protein marker and in each section, lane 2 shows uninduced cells, and lane 3 is the IPTG induced cells after 24 h. The positions of ADO (29.3 kDa) and Fdx, ferredoxin (10.4 kDa) are shown. Figure S4. Residual butanol produced in E. coli and knockout strains engineered to harbour the atoB-TPC7 pathway. Residual butanol produced in the pathway engineered E. coli or knockout strains with wild-type ADO or with the ADOA134F variant enzyme are shown. E. coli and Δahr/ΔyqhD single or double knockout strains were engineered to contain the atoB-TPC7 route (indicated by [file 13068_2015_231_MOESM1_ESM.doc]

**SUPPORTING INFORMATION**

**
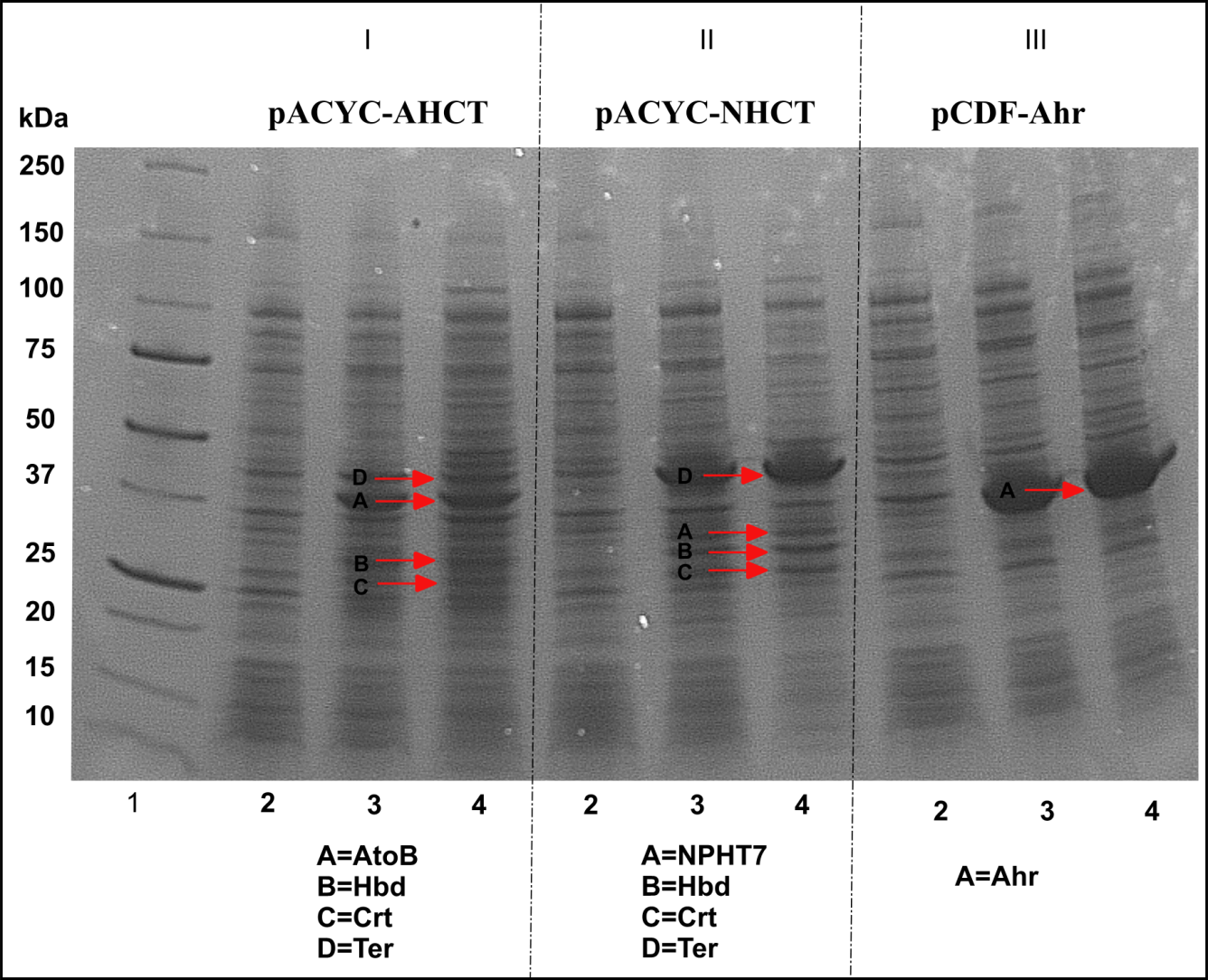
**

**Figure S1** *SDS-PAGE analysis of overexpressed proteins in E. coli cells transformed with plasmids pACYC-AHCT, pACYC-NHCT and pCDF-Ahr*. The SDS-PAGE gel shows IPTG uninduced and induced *E. coli* cells transformed with pACYC-AHCT, pACYC-NHCT and pCDF-Ahr vectors (sections I, II and III, respectively). Lane 1 is the standard protein marker and in each section, lane 2 shows uninduced cells, lane 3 shows IPTG induced cells after 4 hours and lane 4 is the IPTG induced cells after 24 hours. AtoB**,** acetyl-CoA acetyltransferase (molecular weight 40.5 kDa); NphT7**,** acetoacetyl CoA synthase (molecular weight 34.6 kDa); Hbd, 3-hydroxybutyryl-CoA dehydrogenase (molecular weight 30.6 kDa); Crt, 3-hydroxybutyryl-CoA dehydratase (molecular weight 28.2 kDa); Ter, trans-2-enoyl-CoA reductase (molecular weight 43.8 kDa) and Ahr, aldehyde reductase (molecular weight 37.8 kDa) enzymes are marked in the SDS-PAGE. The presence of all proteins expressed from each individual plasmid was confirmed by mass spectrometry analysis of the respective SDS-PAGE bands.

**
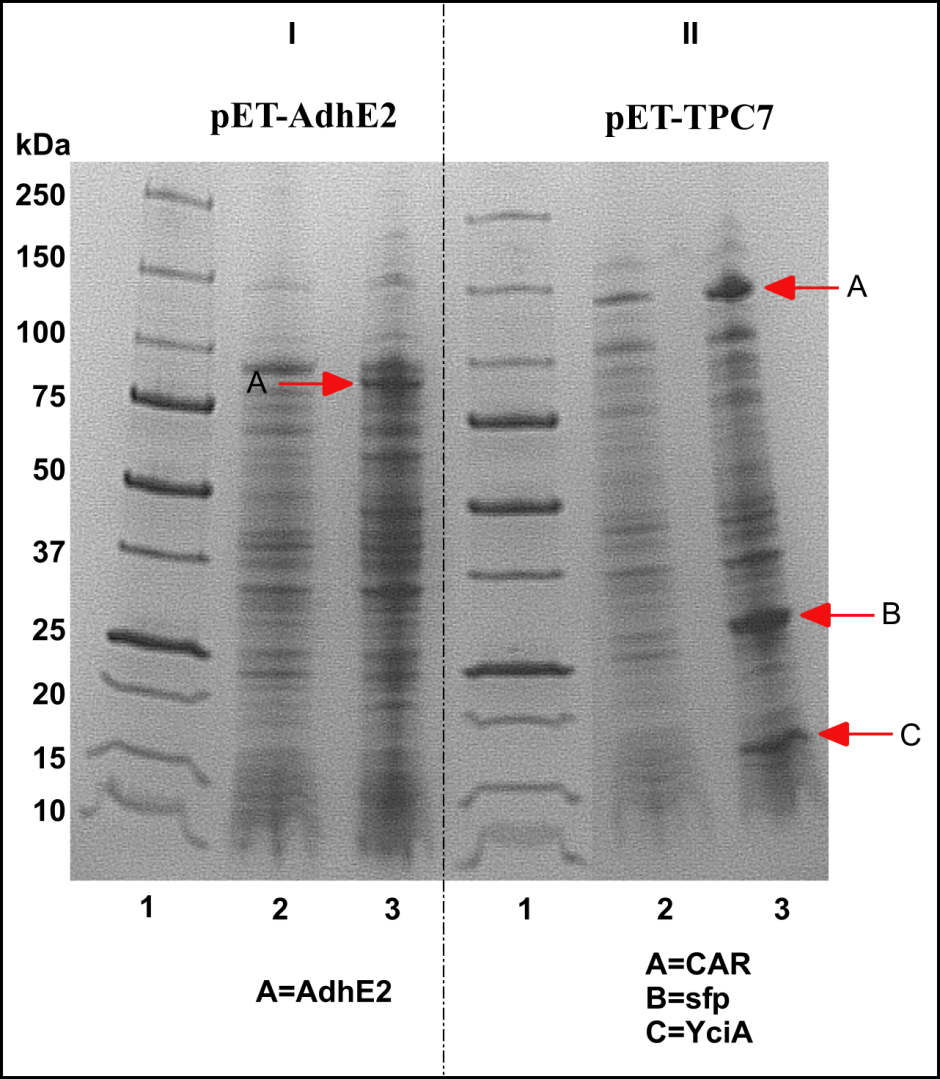
**

**Figure S2.**  *SDS-PAGE analysis of overexpressed proteins in E. coli cells transformed with pET-adhE2 and pET-TPC7 vectors.* The SDS-PAGE gel shows IPTG uninduced and induced *E. coli* cells transformed with pET-AdhE2 and pET-TPC7 vectors (shown in sections I and II, respectively). Lane 1 is the standard protein marker and in each section, lane 2 shows uninduced cells and lane 3 is the IPTG induced cells after 24 hours. The band positions of AdhE2, aldehyde-alcohol dehydrogenase (molecular weight 94.4 kDa); CAR, carboxylic acid reductase (molecular weight 128.9 kDa); sfp (maturation factor phosphopantetheinyl transferase) (molecular weight 26.2 kDa) and YciA**,** acyl-CoA thioester hydrolase (molecular weight 16.7 kDa) are shown. The presence of all proteins was confirmed by mass spectrometry analysis of the respective SDS-PAGE bands.

**
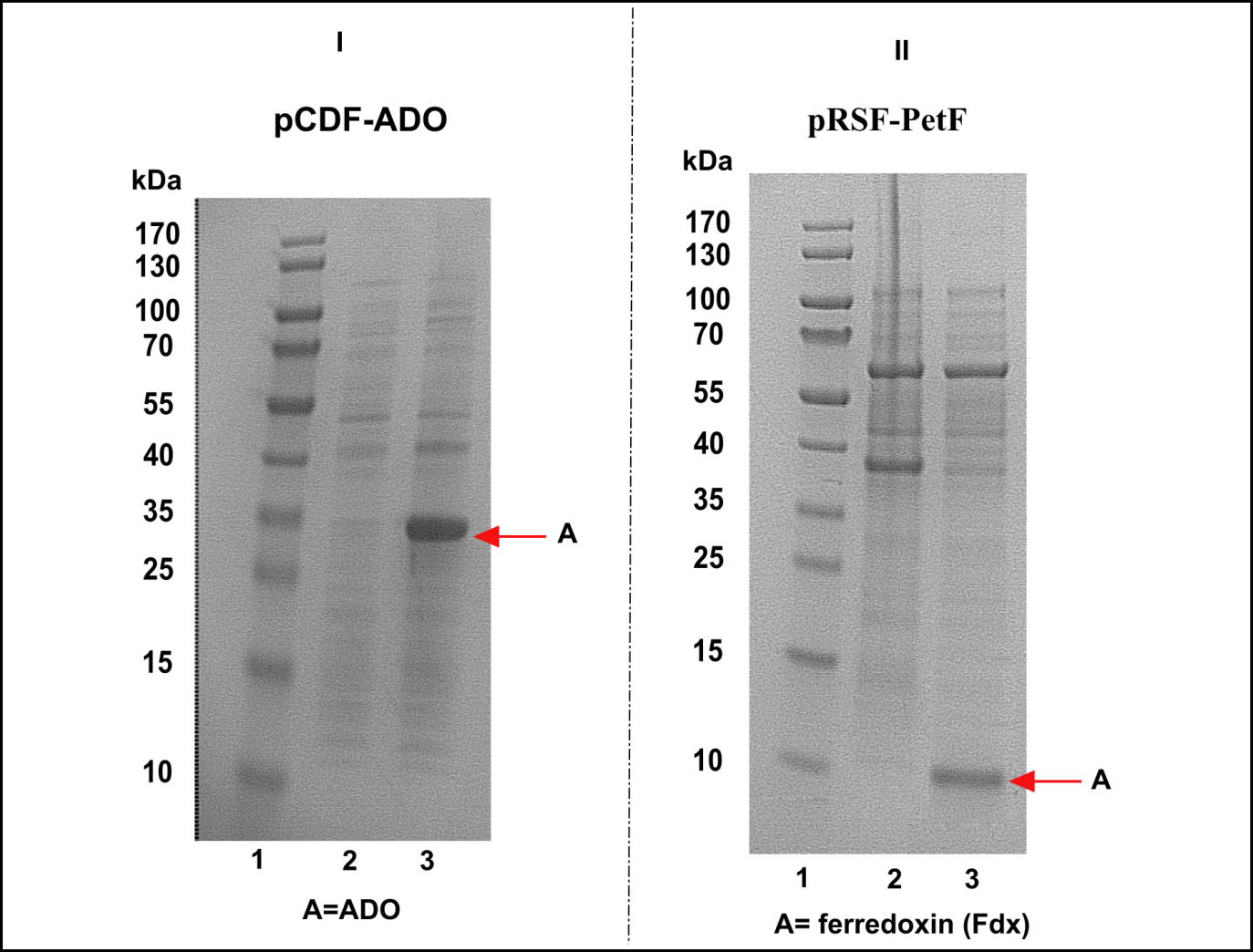
**

**Figure S3.**  *SDS-PAGE analysis of overexpressed proteins in E. coli cells transformed with pCDF-ADO and pRSF-PetF vectors..*The SDS-PAGE gel shows IPTG uninduced and induced *E. coli* cells transformed with pCDF-ADO and pRSF-PetF vectors (shown in sections I and II respectively). Lane 1 is the standard protein marker and in each section, lane 2 shows uninduced cells, and lane 3 is the IPTG induced cells after 24 hours. The positions of ADO, aldehyde deformylating oxygenase (molecular weight 29.3 kDa) and *Fdx*, ferredoxin (molecular weight 10.4 kDa) are shown. The presence of all proteins was confirmed by mass spectrometry analysis of the respective SDS-PAGE bands.

**
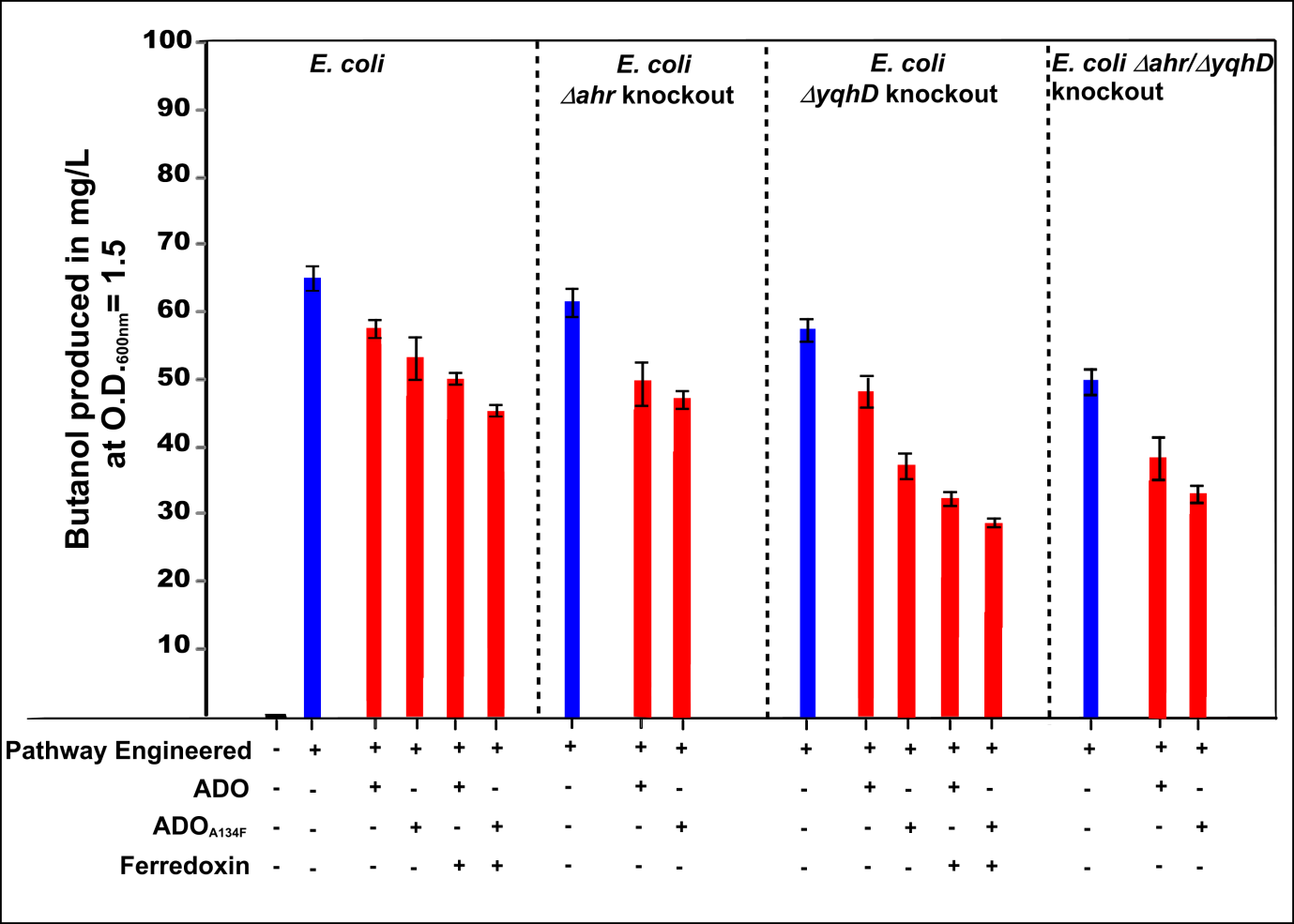
**

**Figure S4** *Residual butanol produced in E. coli and knockout strains engineered to harbour the atoB*-*TPC7* *pathway.* Residual butanol produced in the pathway engineered *E. coli* or knockout strains with wild-type ADO or with the ADO_A134F_ variant enzyme are shown. *E. coli and Δahr/ΔyqhD* single or double knockout strains were engineered to contain the *atoB*-*TPC7* route (indicated by red colour). The effect of wild-type ADO or the ADO_A134F_ variant enzyme with or without *Synechocystis* ferredoxin (Fdx) was also analysed. Detailed protocols for the pathway engineering and residual butanol detection are included in the Materials and Methods section (main manuscript). Error bars are standard deviation (n = 4).

**
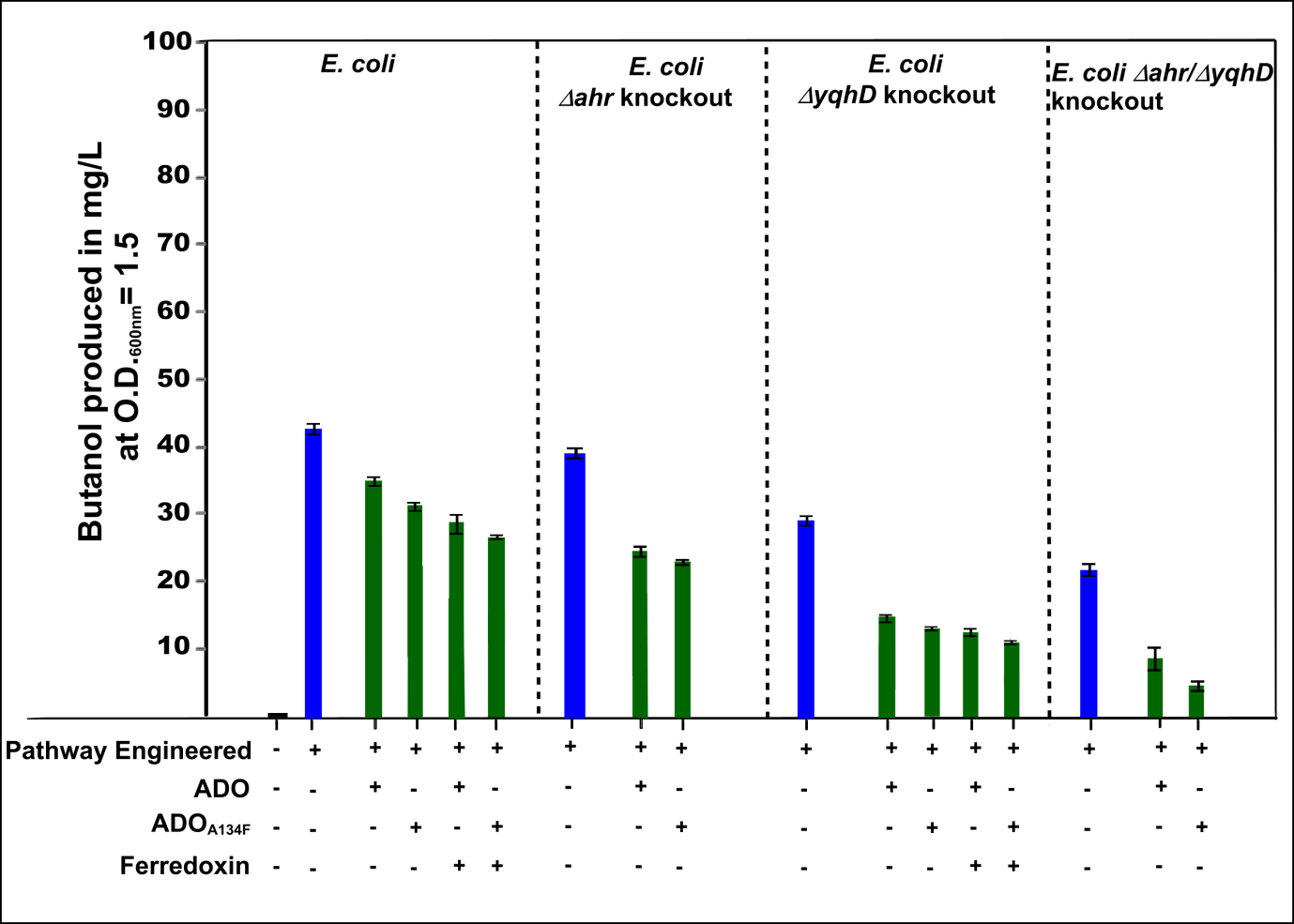
**

**Figure S5** *Residual butanol produced in E. coli and knockout strains engineered to harbour the nphT7*-*TPC7* *pathway*. Residual butanol produced in the pathway engineered *E. coli* or knockout strains with wild-type ADO or with the ADO_A134F_ variant enzyme are shown. *E. coli and Δahr /ΔyqhD* single or double knockout strains were engineered to contain the *nphT7*-*TPC7* route (indicated by green colour). The effect of wild-type ADO or the ADO_A134F_ variant enzyme with or without *Synechocystis* ferredoxin (Fdx) was also analysed. Detailed protocols for the pathway engineering and residual butanol detection are included in the Materials and Methods section (main manuscript). Error bars are standard deviation (n = 4).

**
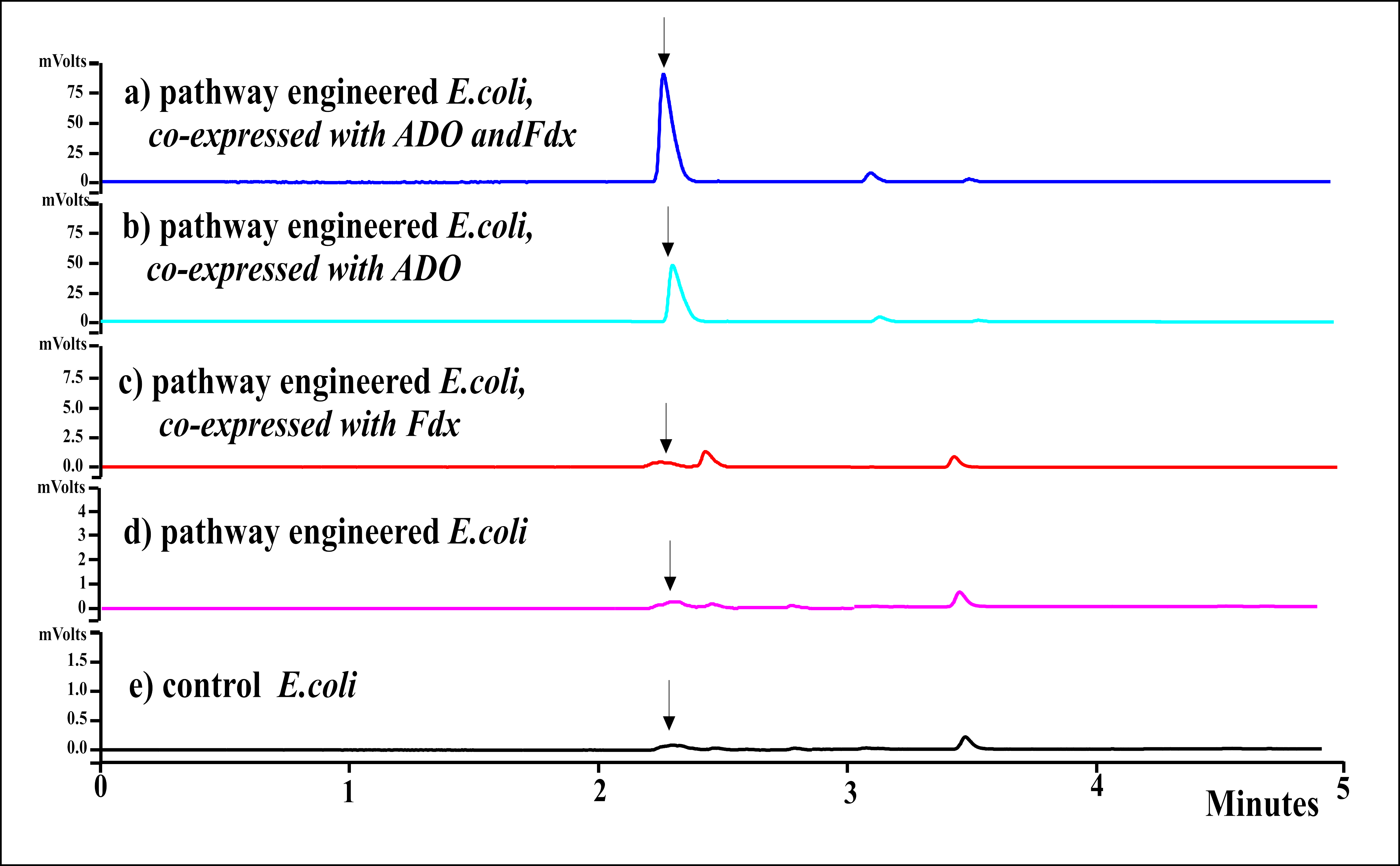
**

**Figure S6** *GC traces and the corresponding propane peak integrated for pathway analysis*. GC traces showing the integrated propane peak for the *AtoB-TPC7* route containing *E. coli BL21* strains coexpressed with ADO and ferredoxin (trace a), *AtoB-TPC7* route containing *E. coli BL21* strains coexpressed with ADO (trace b), *AtoB-TPC7* route engineered in *E. coli BL21* strains coexpressed with ferredoxin in absence of ADO (trace c), *AtoB-TPC7* route engineered in *E. coli BL21* strains (trace d) and control *E. coli BL21* cells without pathway engineering (trace e) are shown.


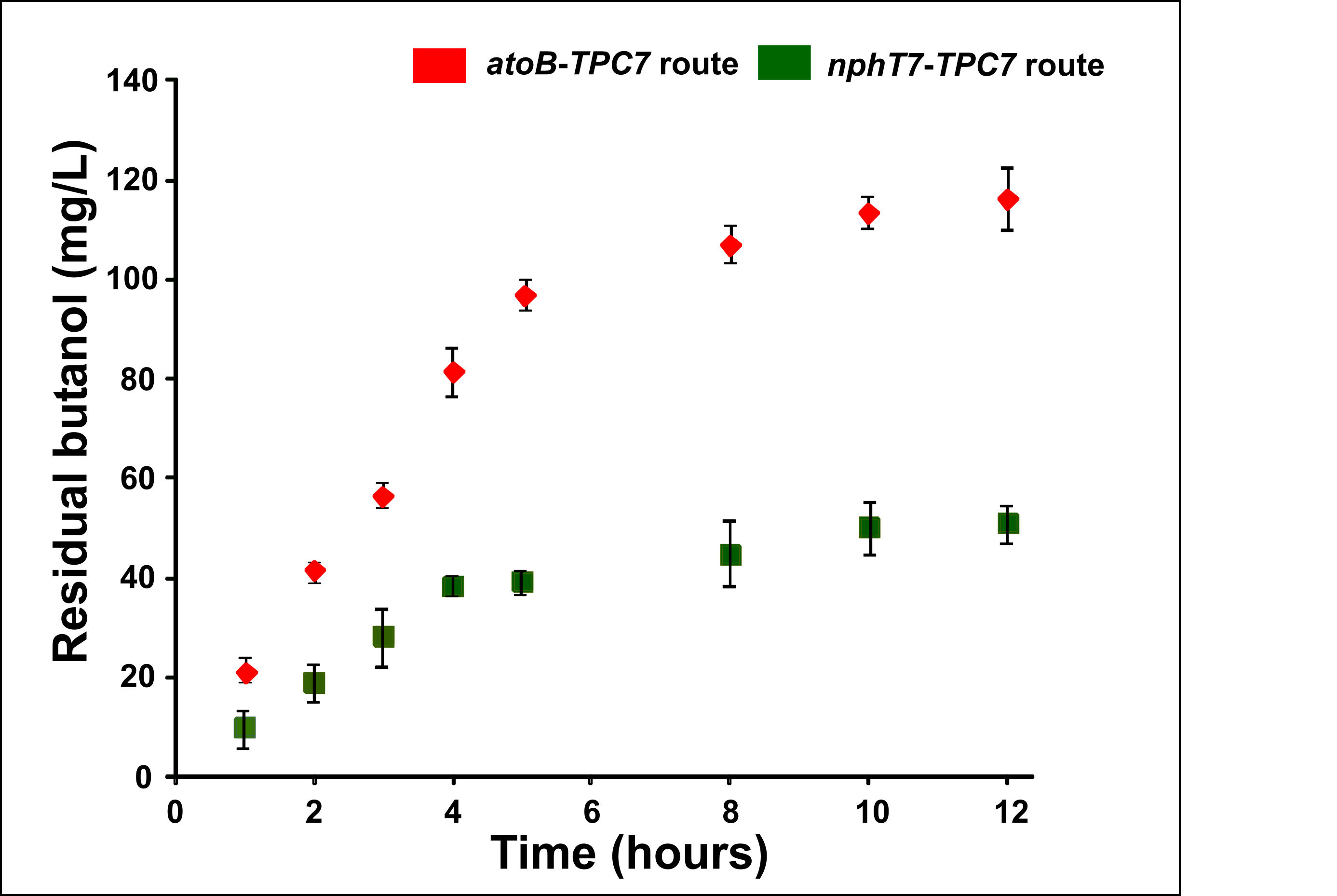


**Figure S7.** Time course for residual butanol accumulation in *large-scale cultures of the best propane-producing pathways.* The *atoB*-*TPC7* route (indicated by red symbols) and *nphT7*-*TPC7* (indicated by green symbols) engineered in *ΔyqhD* knockout cells in the presence of the ADO_A134F_ variant and ferredoxin system were analysed at larger scale. The culture volume was scaled up 400-fold to 200 mL, in a 300 mL flask sealed with airtight rubber septum. The residual butanol in the cultures for 12 hours is shown. Error bars are standard deviation (n=3).
